# Supplementary material for: Conformational Dynamics of the RNA G-Quadruplex and its Effect on Translation Efficiency
Source: Molecules. 2019 Apr 24;24(8):1613. doi: 10.3390/molecules24081613 (PMC6514569; doi:10.3390/molecules24081613)
Supplement: Supplementary file 1 [file molecules-24-01613-s001.pdf]

Supplementary Information

Conformational dynamics of the RNA G-quadruplex and its effect on translation efficiency

Tamaki Endoh[1] and Naoki Sugimoto[1,2]*

[1] Frontier Institute for Biomolecular Engineering Research (FIBER), Konan University, 7-1-20 Minatojima-Minamimachi, Chuo-ku, Kobe 650-0047, Japan

[2] Graduate School of Frontiers of Innovative Research in Science and Technology (FIRST), Konan University, 7-1-20 Minatojima-Minamimachi, Chuo-ku, Kobe 650-0047, Japan

Email: sugimoto@konan-u.ac.jp

Table S1. DNA oligonucleotides for synthesis of G-rich sequence variants

Primers		DNA sequence
mutant B	sense	AATTCAAAGCAGGGCTGGGGCTGGGAGGGGAAAAAAAAG
	antisense	TCGACTTTTTTTTCCCCTCCCAGCCCCAGCCCTGCTTTG
mutant C	sense	AATTCAAAGCAGGGTTGGGGTTGGGAGGGGAAAAAAAAG
	antisense	TCGACTTTTTTTTCCCCTCCCAACCCCAACCCTGCTTTG

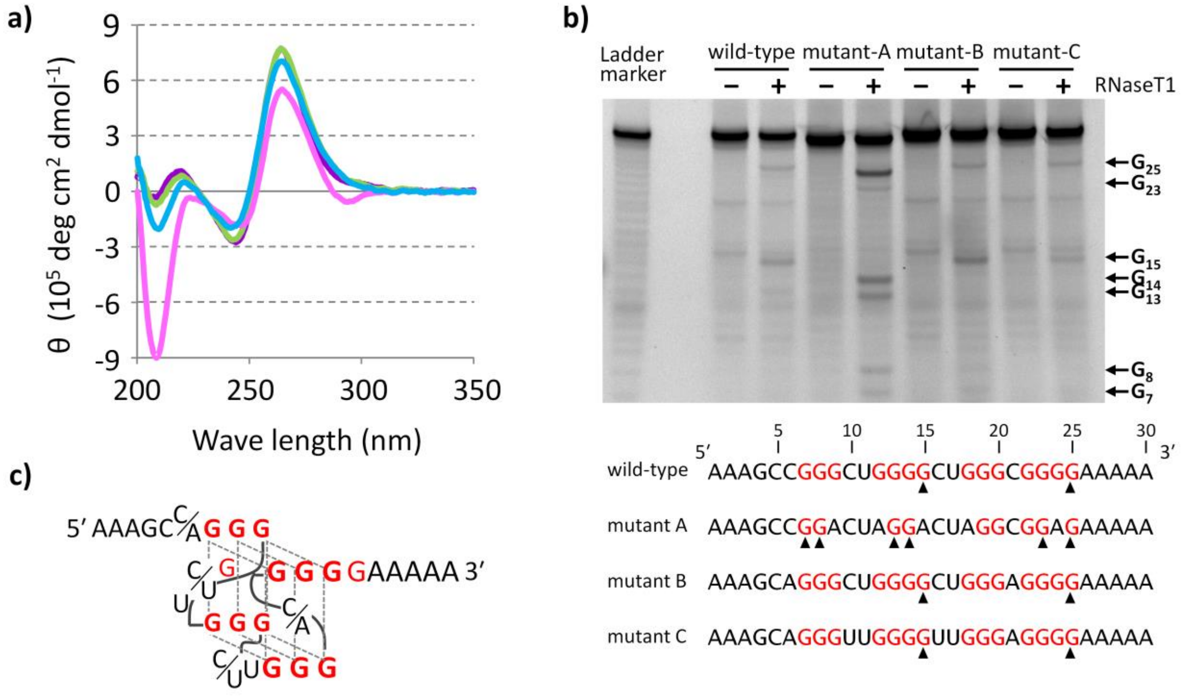

Figure S1. Formation of G-quadruplexes by RNA oligonucleotides. a) CD spectra of wild-type (blue), mutant A (pink), mutant B (green), and mutant C (purple) oligonucleotides in 50 mM Tris-HCl (pH 7.6), 5 mM magnesium acetate, 100 mM KCl, 2 mM spermidine, and 0.01 % (v/v) Tween 20. b) RNase T1 digestion of RNA oligonucleotides. Fluorophore (Alexa-546) labeled oligonucleotides were digested by 0.02 U RNase T1 in a buffer containing 50 mM HEPES-KOH (pH 7.6), 5 mM magnesium acetate, 100 mM potassium glutamate, 2 mM spermidine, and 0.01 % Tween 20. Digested RNA fragments were electrophoresed on a 20 % denaturing polyacrylamide gel at 70°C, and the gel was imaged using 532 nm excitation and 575 nm emission. Ladder marker shows alkaline digested products of wild-type oligonucleotide. Sites of RNase T1 cleavage are indicated below sequences. c) Schematic of parallel RNA G-quadruplex formed by wild-type, mutant B, and mutant C oligonucleotides.

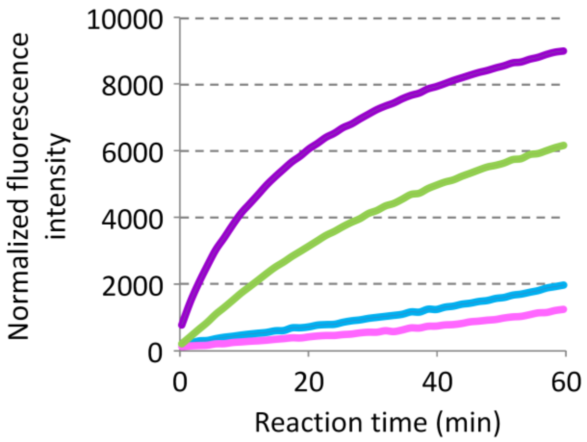

Figure S2. Time course of NMM fluorescence intensities during transcription reactions of wild-type (blue), mutant A (pink), mutant B (green), and mutant C (purple) mRNAs. DNA templates (50 ng/μL) were mixed with T7 RNA polymerase (2 U/μL) in a buffer containing 50 mM HEPES-KOH (pH 7.6), 5 mM magnesium acetate, 100 mM potassium glutamate, 2 mM spermidine, 1 mM rNTPs, 0.01 % Tween20, 0.2 % DMSO, and 10 μM NMM at 37 ˚C. Fluorescence signal of NMM at 610 nm was collected every 77.2 sec by StepOnePlus Real-Time PCR System (Life Technologies), and normalized by subtracting that obtained from reaction mixture without DNA template.

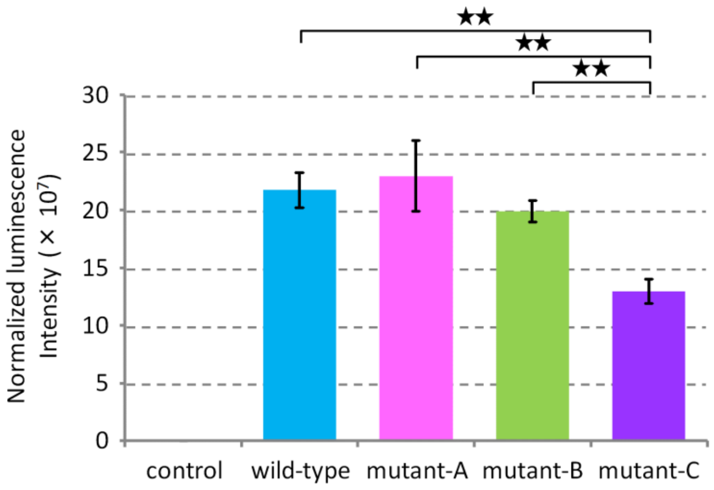

Figure S3. Normalized luminescence intensities of the E. coli lysate cultured in the presence of 2 μM chloramphenicol. Protein expression was induced by 100 μM β-D-1-thiogalactopyranoside in 2× YT medium containing 100 mM potassium glutamate for 1 h. Luminescence signals were normalized by adjusting to an optical density of 600 nm of E. coli cells. Values are expressed as mean ± S.D. of triplicated *E. coli* culturing wells. Asterisks indicate two-tailed P-values for the Student's t-test: *P <0.05 and **P <0.01.

a)

EutE	CCGTGCATTGCCGGGCTGGGGCTGGGCGGGG
GlyQ	AAACCCGACGCTGGGTGCCTGGGGACTGGGCTGGG
GudP	CTTCTTCGGTAAGGGCATCGGGGCGCTGGGTTGGG
RnlA	GGTCCTATGCCAGGGTCAAGGGCTGGGCTTCGGG
YebZ	GCTCGCCGTTCAGGGCGGGTTGATGGGAACGGG

b)

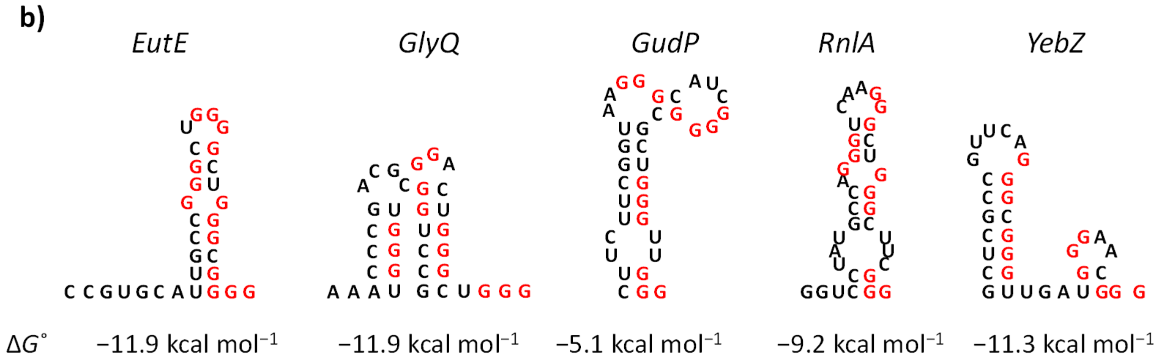

Figure S4. G-rich elements derived from the ORF of the *E. coli* genes. a) Sequences of G-rich elements including 5′ flanking regions. G-rich regions are underlined, and guanine nucleobases expected to be involved in the formation of the G-quadruplex structure are given in red. b) Secondary structures of the G-rich elements predicted using the Mfold program. Thermodynamic stabilities (Δ*G*°) of the secondary structures predicted by the Mfold program are given.
